# Efficacy of neurofeedback as a treatment modality for children in the autistic spectrum

**DOI:** 10.1186/s42269-021-00501-5

**Published:** 2021-02-18

**Authors:** L. Mekkawy

**Affiliations:** Lecturer of Pediatric Neurodisabilities, Department of Medical Studies, Faculty of Postgraduate Childhood Studies, Ain Shams University, Cairo, 2020 Egypt

**Keywords:** Autism spectrum disorder, Theta/beta ratio, Neurofeedback

## Abstract

**Background:**

Neurofeedback (NFB) has been conceded as a convenient measure for both identifying and remodeling neural pliability of brain cells; it is a mean through which participants can have voluntary control on their brain waves being expressed on the EEG. Forty-two autistic children received a NFB therapy aiming at improving their cognitive abilities.

**Results:**

NFB succeeded to decrease children’s high theta/beta ratio by inhibiting theta activity and intensifying beta activity over different sessions. Following therapy, the children’s cognitive functions were found to show comparative improvement compared to pre-treatment assessment on a range of different tasks. Auxiliary improvements were found in their social, thought and attention domains.

**Conclusion:**

These findings propose a basic cognitive function impairment in autism spectrum disorder that can be reduced through specific NFB treatment.

## Background

Autism is usually defined as a behavioral disorder that is mainly distinguished by pervasive impairments in variable aspects of neuro-development such as social interactivity, communication skills and stereotypical behavior bonded with activities (American Psychiatric Association 2013). Until now, no definitive neuro-pathological findings nor any laboratory or performance-based tool gives definitive diagnosis of ASD. Clinical studies in autism have been guided by a number of theories each of them trying to identify the cause: whether cognitive defect, weak central consistency, difficult data processing, the theory-of-mind, neuronal and functional connectivity (Wang et al. 2016).

New research work has identified the neuropathology behind autism as that of a mini-columnopathy. Deficiency in the inhibitory areas that adjoin the cell minicolumn assumes an explanation to the brain excitatory/inhibitory (E/I) imbalance shown in autism. Local E/I interactions design neuronal representations of both motor and sensory changes, as well as cognitive variables, and produce local electroencephalographic (EEG) oscillatory changes (Casanova et al. 2013).

EEG is a commonly used procedure to investigate brain functions in healthy individuals and in those with medical, neurological and psychiatric disorders. EEG is used to examine activity of the brain either during rest, or during evoked brain responses using specific tasks. EEG is either visually inspected by a neurologist (standard EEG) which is able to detect generalized or focal slowing of frequencies in addition to any paroxysmal epileptic activity. Secondly, we have the computer-analyzed reading EEG (C-EEG) which has much advances in assessing the topography (mapping) of the brain changes, detect the deeper cerebral sources of detected abnormalities (source localization) and examine the linearity and nonlinearity of the recorded signal (complexity analysis). (Boutros et al. 2015).

NFB has been conceded as a convenient measure for both identifying and remodeling neural plasticity of brain cells for its noninvasive affinity to change the excess impulses of neural circuits and hence produce a short-term functional reorganization in the neuronal networks in the human brain (Wang et al. 2016).

NFB is a means by which participants can learn to do voluntary control on their brain waves being expressed on the EEG; it has been applied to a variety of different clinical conditions including migraine, epilepsy, attention deficit hyperactivity disorder and traumatic brain injury. During NFB sessions, participants have EEG electrodes attached to their scalp and EEG activity is expressed in the form of sounds or pictures projected on a computer screen and fed back to them automatically through different feedback games. According to this feedback mechanism, children learn to modulate their EEG activity (Karimi and Rostamic 2011).

By operant conditioning of EEG activity, NFB can be considered as an effectual modality to enhance electrophysiological changes of specific cortical area of the brain. NFB treatment is considered one of the successful and salient ways of treatment for attention deficit/hyperactive children (ADHD) (Lofthouse et al. 2010). Given that many children with autism may represent with symptoms and signs of ADHD, studies had attempted to use this new therapy as a treatment multimodality for ASD (Coben 2013; Kouijzer et al. 2010; Linden and Gunkelman 2013).

Many studies reviewed the use of NFB as a treatment multimodality for ASD and some of them proved that the fundamental symptoms of autism can be refined after using this therapy (Coben et al. 2010; Sokhadze et al. 2014).

Clinical recognition of ASD is usually difficult due to the diversity in the presentations and heterogeneity of features, in addition to common comorbidities with other different psychiatric conditions. It is also difficult to diagnose those children because social difficulties and monotonous behaviors are also presented in patients with non-ASD diagnosis such as ADHD, language disorders, learning problems, mental intellectual disability and emotional disorders (Huerta and Lord 2012). This has led to increasing the demand to set up a reliable, valid, inexpensive as well as noninvasive treatment modalities for ASD that can minimize the use and side effects of medications and assess ASD evaluation (Havdahl et al. 2016).

The child behavior checklist (CBCL) is a well-known and very widely used questionnaire filled up by the parents for evaluating behavioral, emotional as well as social problems in children aged 1.5–5 years and 6–18 years (Achenbach and Rescorla 2013). It was established to evaluate a range of variable behavioral problems rather than ASD particularly; recently however, the CBCL has been approved to be useful in ASD within clinical settings (Havdahl et al. 2016).

The CBCL questionnaire measures emotional and behavioral problems in children. It directs seven main domains: aggression, anxiety, depression, attention span, emotional interactions, sleep problems, somatic complaints and withdrawn. CBCL syndrome domain *t*-scores were categorized as clinical (60 or greater) and non-clinical (< 60) (Moody et al. 2017).

This present study worked out to design a technique to monitor EEG activity in autistic children and interpret the different changes during NFB sessions in those children. The study is one of the approaches aiming at understanding the correlation between EEG brain waves activity and NFB training in ASD population. In addition to the practical evaluation of NFB as a modality tool of treatment for ASD, the current study aimed to understand the cognitive and neural processes that underlie NFB improvements in the core of ASD.

## Methods

### Participants

The present study is a randomized clinical trial that was conducted on 50 patients who were following up at the outpatient clinics of our center of special needs. Only 42 patients (33 males and 9 females) managed to fulfill the study (Table 1). Children included were between 6 and 18 years old (Table 2), an IQ-score of 70 and above (Table 3), being diagnosed as autism according to DSM-V by our child psychiatrist. Children under medical treatment, those with a history of severe brain insult, as well as children with epilepsy were excluded. An informed consent was signed from all parents/caregivers before the start of the therapy. The protocol of the study was approved by the scientific research ethical committee of our faculty.

**Table 1:** Patient’s sex frequency

Age	Frequency	Percent
Male	33	78.6
Female	9	21.4
Total	42	100

**Table 2:** Patient’s age frequency

Age	Frequency	Percent
6.0	6	14.3
6.5	4	9.5
7.0	9	21.4
7.5	2	3.1
8.0	6	14.3
8.5	1	2.4
9.0	5	11.9
9.5	1	2.4
10.0	2	3.1
10.5	1	2.4
11.0	4	9.5
12.5	1	2.4
Total	42	100

**Table 3:** Patient’s IQ test score

IQ	Frequency	Percent
70.0	5	11.9
72.0	5	11.9
74.0	7	16.7
76.0	1	2.4
77.0	4	9.5
78.0	2	4.8
80.0	8	19.0
82.0	3	7.1
84.0	2	4.8
86.0	1	2.4
88.0	3	7.1
90.0	1	2.4
Total	42	100.0

### Procedures

#### NFB protocol and data collection

EEG recording was performed and NFB sessions were digitalized using the digital cortical scan device Mitsar 201 (Mitsar Medical Diagnostic Equipment, Russia); head electrodes were placed according to the international 10–20 system using a 19 electrode cap with a ground electrode at FZ and ears linked as references. The impedance of all electrodes was adjusted < 10 kΩ; all input signals were filtered between 0.5 and 50 Hz. EEG recording was collected while children were seated upright in a straight back chair during two conditions each lasted 20 min: eyes opened and eyes closed without any sedation. Finally, EEG was inspected visually to verify removal of any artifacts.

Each child received 40 sessions of NFB treatment, given 3 times a week; at the beginning of each session, a single-channel EEG recording was done where band ranges for theta and beta were set at 4–7 Hz (theta), and 13–21 hz (beta) registered at FZ, CZ in the eyes opened condition. Theta/beta ratio (TBR) coefficient was calculated by dividing the activity of the slower band by the activity of the faster frequency band.

#### The child behavior checklist (CBCL)

Simultaneous with brain waves data collection, data concerning the behavioral ratings using the CBCL questionnaire were done.

### Statistical methods

Obtained data were collected, analyzed, coded and finally tabulated using SPSS® computer package version 12.0. Numerical variables were presented as mean and standard deviation, while categorical variables were presented as frequency and percentage. Every given dependent variable represented on the electroencephalogram over 40 sessions of NFB was scanned using linear regression analyses, and the mean values of those variables lasting from session 1 to session 40 of the NFB course together with the pre- and post-NFB behavioral assessment using CBCL were compared with the Wilcoxon paired test. Spearman correlation analysis was applied for individual electroencephalogram variables, NFB signals and behavioral assessments collected using CBCL questionnaire.

## Results

### Demographic analysis:

The sample consists of 42 patients, and its demographic characteristics are classified into three main characteristics which are: age, sex and IQ test score.

### Descriptive analysis

The following table presents the descriptive analysis for the sample variables which are: first TBR, last TBR, CBCL pre, CBCL post, social pre, social post, thought pre, thought post, attention pre, attention post, first T score and last T score.

### Correlation matrix

The following matrix presents Spearman coefficient of correlation between TBR, last TBR, CBCL pre and CBCL post as the four variables are not normally distributed (Table 4).

**Table 4:** Correlation matrix between study variables

Variable	First TBR	Last TBR	CBCL Pre	CBCL post
first TBR	1.000			
*p* value	–			
last TBR	0.516**	1.000		
*p* value	0.000	–		
CBCL pre	0.752**	0.492**	1.000	
*p* value	0.000	0.001	–	
CBCL post	0.448**	0.528**	0.505**	1.000
*p* value	0.003	0.000	0.001	–

### Wilcoxon paired sample test

Wilcoxon paired sample test is a nonparametric test for testing the difference between means for the same sample after applying a certain procedure. Wilcoxon paired sample test applied to compare between the means of (first TBR and last TBR), (CBCL pre and CBCL post) and (first T score and last T score).

## Discussion

The present study assisted in the evaluation of using NFB training protocol (using theta/beta ratio as a prognostic tool) as a treatment modality for autistic children. It was hypothesized that reduction of theta power improves childrens’ cognitive capacities/functions. Consonant with the study’s prediction, children included in this study managed to improve their performance over a range of variable cognitive processes after NFB training. Furthermore, these findings provided evidence supporting that NFB may be considered as a valuable treatment modality for children with ASD.

At a neurophysiological level, NFB training was able to increase low beta power (12–15 Hz) and reduce theta power (4–7 Hz) in all autistic participants. Theta/beta ratios showed marked regression over 40 sessions of NFB, changes in EEG amplitude following NFB training mainly affected 3 main areas in the brain, regressive changes were mostly seen in FZ, F4 (areas of socialization and communication) followed by CZ (area of activity) and lastly the temporal areas T3, T4 (areas of emotions). In addition *t*-score showed that the ratios decreased statistically throughout the whole course of treatment (Table 5).

**Table 5:** Study variables descriptive analysis

Variable	*N*	Minimum	Maximum	Mean	SD
First TBR	42	5.0	11.0	8.081	1.3399
Last TBR	42	2.8	8.0	4.652	1.4980
CBCL Pre	42	76.0	120.0	94.357	9.0576
CBCL post	42	35.0	108.0	60.357	20.6218
Social pre	42	14.0	20.0	16.571	2.0380
Social post	42	6.0	19.0	10.190	3.1872
Thought pre	42	7.0	20.0	14.524	2.9734
Thought post	42	4.0	18.0	7.452	3.7168
Attention pre	42	14.0	19.0	16.452	1.5174
Attention post	42	7.0	17.0	10.905	2.6943
First T score	42	72.0	79.0	75.214	1.6008
Last T score	42	58.0	78.0	66.690	5.8705

**Fig. 1:**
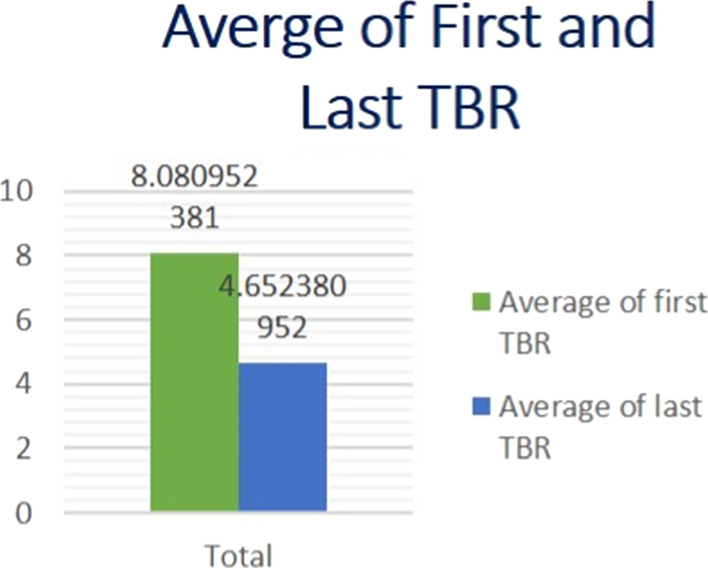
Average first and last TBR

According to the results of the current study, statistical significant (*P* < 0.001) improvement in TBR was found throughout the whole treatment course (Table 6, Fig. 1).

**Table 6:** Wilcoxon test for mean difference of TBR

First TBR mean	Last TBR mean	Mean difference	Standard error	T-statistic	df	*p* value
8.081	4.652	3.428571	0.2357504	14.5432	41	0.000

Our findings are concomitant with the research work of Coben and Padolsky (2007) who established significant changes in children’s brain waves coherence after NFB training they found a decline in seventy six percent of the intervention group.

Similarly, Wang et al. (2016) found that the prefrontal regression rate in the TBR was vigorous across treatment. In addition, Kouijzer et al. (2009) proved theta reduction in his autistic children sample and that NFB caused changes in their brain waves which improved their social behavior, communication interactions and synchronization.

At the intellectual level, NFB treatment was theorized to enhance the cognitive functions of children with ASD. Results indicated significant improvement in attentional span, cognitive flexibility in addition to social interaction for children in the studied group where their parents reported improvement on the CBCL questionnaire subscales.

**Fig. 2:**
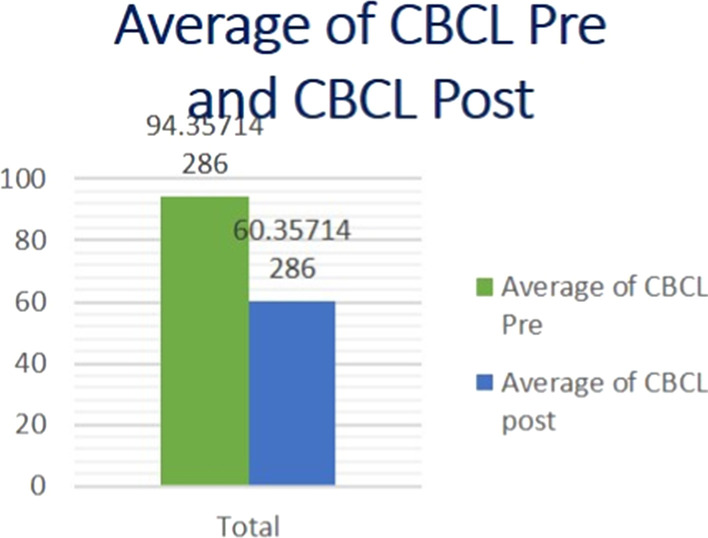
Average pre- and post-CBCL

**Fig. 3:**
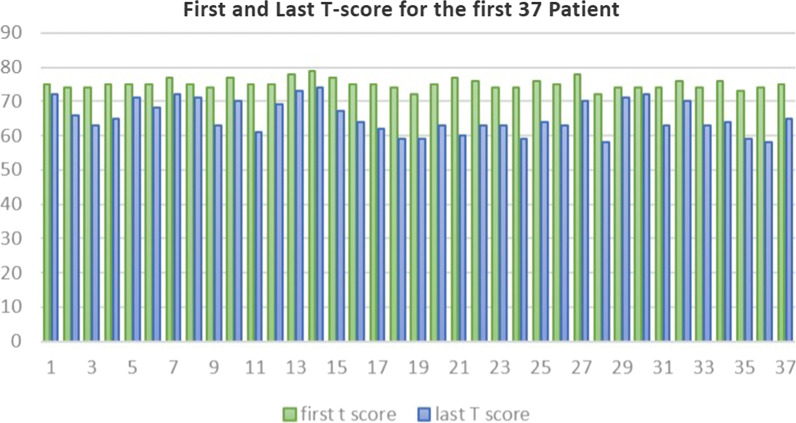
First and last TBR for the first 37 patients

**Fig. 4:**
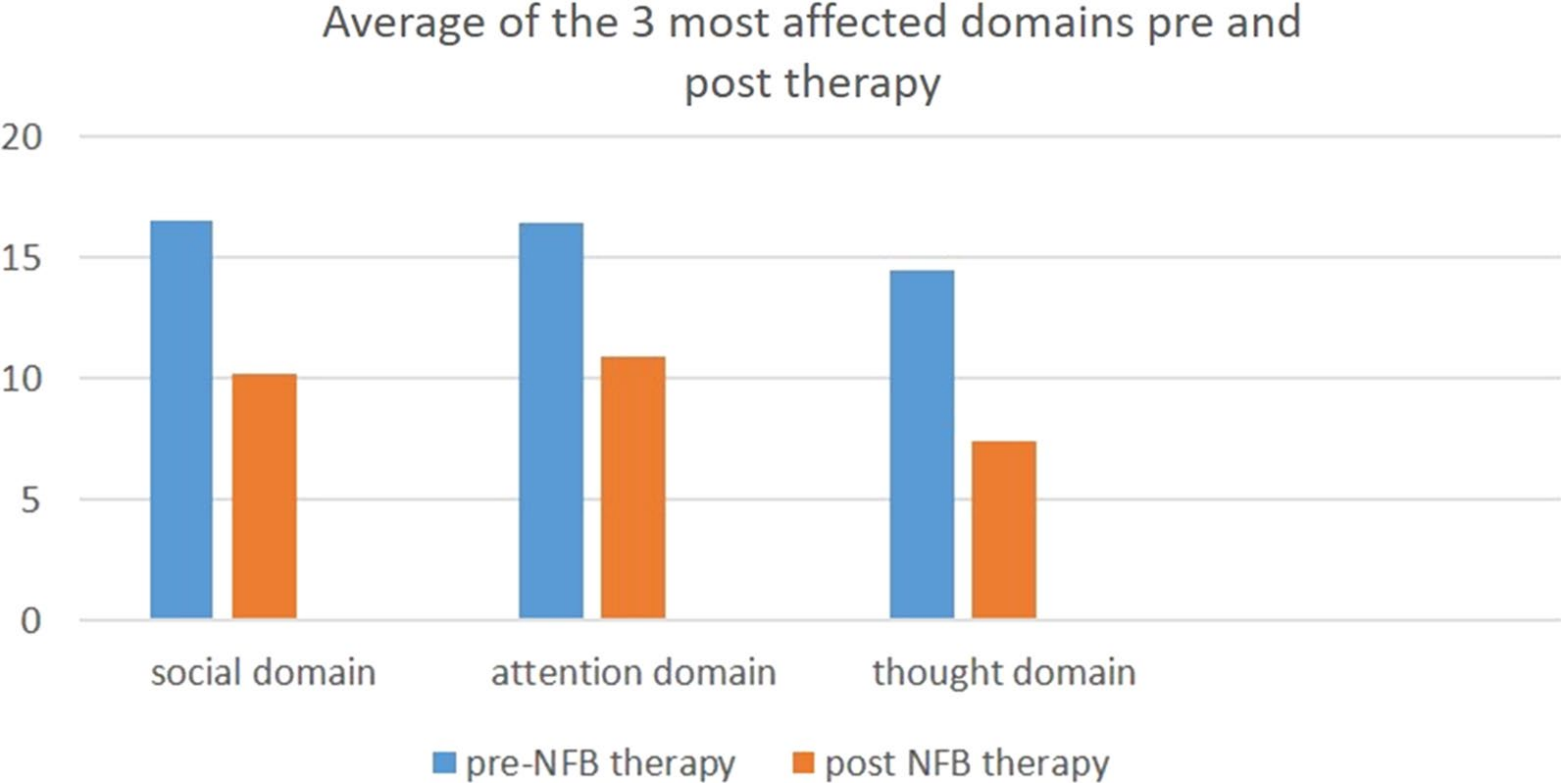
First and last T score for the last 5 patients

**Fig. 5:**
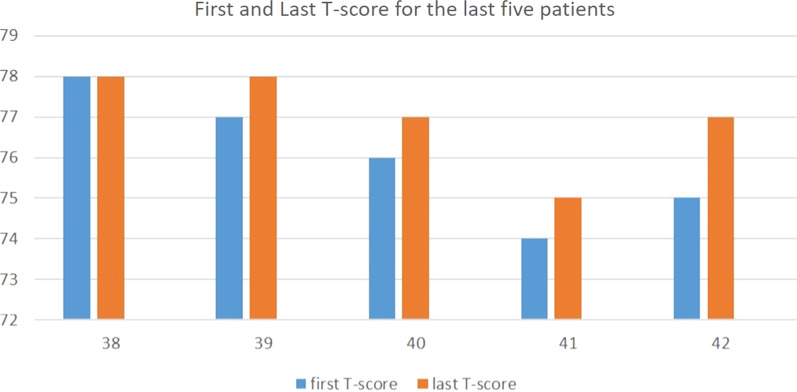
The 3 main domains of CBCL affected by NFB

According to our study, (Tables 7, 8, Fig. 2), statistically significant improvement of CBCL questionnaire and reduction of the total t score of the test was noted. The first 37 patients (Fig. 3) showed reduction in their total *t*-score reflecting improvements in three main domains: social interaction, thought and attention span (Fig. 4). However, only five patients did not show any improvement (Fig. 5); those five patients were males in gender with mean age (10.7) and mean IQ level (74.8). This raises the idea that the earlier the intervention takes place, the better impact and outcome can be found for both the children and their families.

**Table 7:** Wilcoxon test for mean difference of CBCL

CBCL pre mean	CBCL post mean	Mean difference	Standard error	T-statistic	df	*p* value
94.357	60.357	34	2.739143	12.4126	41	0.001

**Table 8:** Wilcoxon test for mean difference of T score

First T score mean	Last T score mean	Mean difference	Standard error	T-statistic	df	*p *value
75.214	66.690	8.532809	0.7899176	10.708	41	0.000

Our results are concomitant with studies of Jarusiewicz (2002), Coben and Padolsky (2007), and Pineda et al. (2008), who proved to find wide range of improvement in social interactions, behavior and verbal communication NFB treatment.

Congedo et al. (2009) listed significant upgrading in social interchanges, as well as stereotyped and monotonous behavior after NFB treatment.

Kouijzer et al. (2009) proved significant improvement in autistics attentional spam level, intellectual pliability as well as setting goals compared to the control group.

## Conclusion

To conclude, applying a NFB treatment protocol to a group of ASD children proved to be reasonably successful. NFB therapy showed remarkable improvements in children’s cognitive abilities. These findings advocate a correlation between amplified TBR training in these children and hypo-activation of the anterior cingulate cortex as a possible neural core problem for this impairment.

This study faced several limitations. The enlistment of NFB therapy was applied only to high functioning autistic patients; thus, results cannot represent low functioning children with ASD. Also following up for 40 sessions or more was extremely difficult due the current circumstances of COVID-19 pandemic.

This study focused on interpretation of electroencephalogram activity that was interpreted using specially customized software aiming at exploring the dynamics of brain waves activity during the NFB treatment in autism. Detailed demographic information’s and medication status were not fully analyzed. To encourage using NFB as a treatment modality for autistic children and its scientific reasoning, further different study designs, larger representative sample, more intensive baseline, treatment as well as follow-up assessments will be recommended.

## Data Availability

Available upon request.

## Abbreviations

ACC: Anterior cingulate cortex
ADHD: Attention deficit hyperactivity disorder
ASD: Autism spectrum disorder
CBCL: Child behavioral checklist
DSM: Diagnostic and statistical manual of mental disorders
EEG: Electroencephalogram
I/E: Inhibitory excitatory
IQ: Intelligence quotient
TBR: Theta beta ratio
